# Pilot trial of remote monitoring to prevent malnutrition after hepatopancreatobiliary surgery

**DOI:** 10.1186/s40795-021-00487-3

**Published:** 2021-12-09

**Authors:** Kelvin Allenson, Kea Turner, Brian D. Gonzalez, Erin Gurd, Sarah Zhu, Nicole Misner, Alicia Chin, Melissa Adams, Laura Cooper, Diana Nguyen, Samer Naffouje, Diana L. Castillo, Maria Kocab, Brian James, Jason Denbo, Jose M. Pimiento, Mokenge Malafa, Benjamin D. Powers, Jason B. Fleming, Daniel A. Anaya, Pamela J. Hodul

**Affiliations:** 1 Department of Gastrointestinal Oncology, Moffitt Cancer Center, Tampa, Fl USA; 2 Department of Health Outcomes and Behavior, Moffitt Cancer Center, Tampa, FL USA; 3 University of South Florida Morsani College of Medicine, Tampa, Fl USA; 4 Department of Nutrition Therapy, Moffitt Cancer Center, Tampa, Fl USA

**Keywords:** Mobile app, Digital health, Surgery, Hepatopancreaticobiliary, Nutrition, Malnutrition, Cancer

## Abstract

**Background:**

Patients undergoing hepatopancreatobiliary (HPB) surgery, such patients with pancreatic, periampullary, and liver cancer, are at high risk for malnutrition. Malnutrition increases surgical complications and reduces overall survival. Despite its severity, there are limited interventions addressing malnutrition after HPB surgery. The aim of this pilot trial was to examine feasibility, acceptability, usability, and preliminary efficacy of a remote nutrition monitoring intervention after HPB surgery.

**Methods:**

Participants received tailored nutritional counseling before and after surgery at 2 and 4 weeks after hospital discharge. Participants also recorded nutritional intake daily for 30 days, and these data were reviewed remotely by registered dietitians before nutritional counseling visits. Descriptive statistics were used to describe study outcomes.

**Results:**

All 26 patients approached to participate consented to the trial before HPB surgery. Seven were excluded after consent for failing to meet eligibility criteria (e.g., did not receive surgery). Nineteen participants (52.6% female, median age *=* 65 years) remained eligible for remote monitoring post-surgery. Nineteen used the mobile app food diary, 79% of participants recorded food intake for greater than 80% of study days, 95% met with the dietitian for all visits, and 89% were highly satisfied with the intervention. Among participants with complete data, the average percent caloric goal obtained was 82.4% (IQR: 21.7).

**Conclusions:**

This intervention was feasible and acceptable to patients undergoing HPB surgery. Preliminary efficacy data showed most participants were able to meet calorie intake goals. Future studies should examine intervention efficacy in a larger, randomized controlled trial.

**Trial registration:**

Clinicaltrials.gov. Registered 16 September 2019, https://clinicaltrials.gov/ct2/show/NCT04091165.

## Introduction

Hepatopancreaticobiliary (HPB) surgeries have increased over the past two decades in the U.S. [1–3]. Part of this growth has been driven by a rise in pancreatic, liver, and periampullary cancers, which are treated through HPB surgery [4–6]. HPB surgery is associated with extensive morbidity. After HPB surgery, about a quarter of patients experience serious complications, such as infections and blood clots [7–9], and about 15–20% of patients are readmitted to the hospital within 30 days of HPB surgery [10, 11]. One of the most challenging aspects of patient management following HPB surgery is malnutrition, or the inadequate uptake or absorption of nutrients for maintaining one’s health [12]. Up to 40% of patients undergoing HPB surgery experience malnutrition 30 days after surgery [13–16]. There is a critical need to develop interventions that reduce malnutrition among patients undergoing HPB surgery.

There are several reasons why malnutrition is common after HPB surgery. HPB surgeries change the digestive system, often causing symptoms that interfere with food intake and malabsorption of nutrients [17–19]. Patients can also experience complications that further interfere with nutrition, such as pancreatic exocrine insufficiency (i.e., lack of digestive enzymes), endocrine insufficiency (i.e., glucose intolerance), and delayed gastric emptying due to impaired motor function of the stomach [20–24]. Left untreated, malnutrition can have devastating consequences, such as impaired immune functioning, loss of lean body mass, increased risk for infection, decreased receipt of adjuvant therapy, and lower survival [13, 25–27]. Despite its severity, there is limited evidence about how to treat malnutrition after HPB surgery [28, 29].

Nutritional counseling is effective for preventing malnutrition among patients with cancer, but this strategy has been underused among HPB surgery patients [28, 30–32]. To prevent malnutrition after surgery, patients must make significant dietary changes, such as eating small and frequent meals, and may require supplements or insulin to manage pancreatic exocrine or endocrine insufficiency. Despite the importance of dietary self-management, patients undergoing HPB surgery are often discharged from the hospital without any dietitian support and report feeling overwhelmed and underprepared to manage their nutrition [33, 34]. For example, *fewer than a quarter of* pancreatectomy *patients receive any dietitian support* post-surgery [31, 35], even though this is recommended by clinical guidelines [20]. Prior studies suggest that nutrition counseling is enhanced when patients are given tools to track their food intake, an approach known as dietary self-monitoring [36–38]. Dietary self-monitoring or recording daily consumption of foods and beverages is an evidence-based approach for behavioral weight loss interventions [38]. In order for dietary self-monitoring to work, patients must adhere to daily tracking of their food intake [39]. However, self-monitoring can be burdensome and decline over time, especially when using paper food logs [40]. Therefore, digital tools may be optimal for promoting adherence to dietary self-monitoring among HPB surgery patients. However, this strategy has not been tested in HPB surgery patients.

To address this gap, this pilot trial examined the feasibility, acceptability, usability, and preliminary efficacy of an intervention to provide remote nutritional monitoring for patients after HPB surgery. The intervention combines two evidence-based approaches: 1) nutrition counseling from a dietitian delivered, and 2) dietary self-monitoring through a mobile application (app). Findings from this pilot study could support future studies to test intervention efficacy in a larger trial and to scale up the intervention to additional patients at-risk for malnutrition should the intervention prove efficacious.

## Methods

### Study design

A single-arm, pilot trial was conducted at Moffitt Cancer Center in Tampa, FL.

### Study population

Patients with operable malignant or pre-malignant disease of the liver or pancreas, with treatment plan for neoadjuvant chemotherapy or chemoradiation or up-front resection were prospectively identified, consented, and followed through the Pancreatic and Hepatobiliary clinics between August 2019 to May 2020. Eligible patients were 18 years or older with biopsy-proven malignancy or those with pre-malignant diagnoses undergoing planned pancreatectomy or hepatectomy. Patients were also required to own a smartphone, be willing to use a mobile app for the tracking of post-operative nutritional intake, answer nutrition-related questionnaires, and be able to speak English. Patients were excluded if they were deemed ineligible for surgery or required parenteral or enteral nutrition. Patients provided written consent to participate in the study prior to surgery. The target sample size was 20 patients, which was deemed sufficient to test feasibility. The Advarra Institutional Review Board reviewed and approved all study activities, and the study was registered at ClinicalTrials.gov (NCT04091165).

### Dietitian counseling and mobile app use

Patients received dietary counseling by a registered dietitian with oncology experience in the pre-, peri-, and post- operative setting. Before surgery, patients were screened for malnutrition using a validated screening tool, the Patient-Generated Subjective Global Assessment [41]. Information from the malnutrition screening tool was used to individualize calorie goals for each patient. The dietitian set calorie goals based on the Mifflin St Jeor equation with a 1.4–1.5 activity factor depending on malnutrition status. The dietitian also met with the patient after surgery while the patient was hospitalized to review dietary goals and provide food intake instructions. After hospital discharge, the dietitian met with the patient at 2 and 4 weeks to provide counseling on how to increase calorie intake to meet goals (e.g., calorie-dense foods, oral nutrition supplements) and strategies for managing nutrition-related side effects.

In addition to dietary counseling, patients were provided with a mobile app, the MyPlate mobile app (Leaf Group Ltd.; Santa Monica, CA) at the initial dietitian visit. The dietitian helped patients download and enter nutrition goals into the app. Patients were asked to record all dietary intake using the app for a period of 30 days after hospital discharge. The app calculates daily caloric intake based on entered data. Entry of any food items in a day was defined as use of the app for the day. The dietitian remotely monitored patients’ food intake data and used the information to guide nutrition counseling visits (e.g., provided feedback on caloric goal obtainment).

### Study measures

#### Feasibility and acceptability

Feasibility was defined as recruitment, retention, and app use rates. The benchmark for recruitment was ≥50% of eligible patients consenting to participate in the study. The benchmark for retention was ≥70% of participants retained over the study period and ≥ 70% submitting complete food intake data for ≥80% of study days. Acceptability was defined as patient engagement and satisfaction with the intervention. The benchmark for patient engagement was ≥70% of participants attending all three dietitian visits (i.e., baseline, 2 weeks, 4 weeks). Intervention satisfaction was measured using an item assessing participants’ level of agreement with the statement, “I am satisfied with using the mobile app to help follow my nutrition plan.” The item was measured using a 5-point Likert scale ranging from completely disagree to completely agree. The benchmark for acceptability was ≥70% of participants completing agreeing with the statement.

#### Usability

Usability was defined as self-reported ability to learn and use the app. Usability was measured by assessing participants’ level of agreement with three statements: “The mobile app was easy to learn”; “Navigating the mobile app was clear and understandable”; and “It was easy to log meals on the mobile app.” The items were measured using a 5-point Likert scale ranging from completely disagree to completely agree. The benchmark for usability was ≥70% of participants agreeing or completing agreeing with all three statements.

#### Caregiver involvement

We asked participants whether their informal caregiver (e.g., spouse or family member) assisted the participant with logging their food intake.

#### Preliminary efficacy

Preliminary efficacy was defined as the percent of caloric goal obtained over a 30-day period. This was determined by calculating the average calories consumed per day as documented in the MyPlate Calorie Counter mobile app by the calorie goal determined by the dietitian.

## Statistical analyses

Descriptive statistics were used to describe study outcomes. For categorical variables, sample size and percent are reported. For continuous variables, median and interquartile range (IQR) is reported.

## Results

### Sample characteristics

Of the 26 participants who consented prior to surgery, seven did not receive surgery (e.g., cancer metastasis). The median age of study (*n* = 19) participants was 65 (IQR: 15) years (Table 1). Most participants were White (84.1%) and female (52.6%). The median Charlson comorbidity score was 6 (IQR: 2). The median body mass index (BMI) was 26.2 (IQR: 6.8). Most patients received a pancreatectomy (78.9%) while fewer underwent a hepatectomy (21.1%). PG-SGA scores measuring malnutrition risk ranged from 0 to 16. Most patients received a score of 2–3 (36.8%) indicating a recommendation for dietitian intervention or a score of 4–8 (36.8%) indicating a dietitian intervention is required. Some patients (15.9%) received a score of ≥9 indicating a critical need for intervention while fewer patients (10.5%) received a score of 0–1 indicating no immediate intervention was needed. All patients indicated that they had used a mobile application previously.

**Table 1:** Participant characteristics

Characteristic	***N*** = 19
Age, median (IQR)	65.0 (15.0)
Gender, n (%)	
Female	10 (52.6)
Male	9 (47.4)
Race and Ethnicity, n (%)	
White	16 (84.1)
Non-Hispanic Black	1 (5.3)
Asian	1 (5.3)
Hispanic	1 (5.3)
Charlson Comorbidity Index, median (IQR)	6.0 (2.0)
Body Mass Index, median (IQR)	26.2 (6.8)
ASA Physical Status, n (%)	
Score 2	5 (26.3)
Score 3	14 (73.7)
Surgery type, n (%)	
Hepatectomy	4 (21.1)
Pancreatectomy	15 (78.9)
PG-SGA Score	
0–1 (No immediate intervention)	2 (10.5)
2–3 (Dietitian intervention recommended)	7 (36.8)
4–8 (Dietitian intervention required)	7 (36.8)
≥ 9 (Critical need for dietitian and clinician intervention)	3 (15.9)

*IQR* Interquartile range, *ASA* American Society of Anesthesiologists, *PG-SGA* Patient-generated Subjective Global Assessment

### Feasibility and acceptability

Of the 26 patients approached to participate in the study, all 26 consented to participate, a 100% recruitment rate. Seven participants were excluded after consent for failing to meet inclusion criteria including 1) surgery was aborted due to intra-operative finding of metastatic disease or cirrhosis precluding safe resection or 2) patient required parenteral or enteral nutrition after surgery. The remaining eligible 19 patients were retained over the study period, a 100% retention rate. The percent of patients that submitted food intake data for at least 80% of study days was 15/19 (78.9%). The percent of patients who attended all three dietitian visits was 18/19 (94.7%). Nearly all participants who completed the exit survey 16/18 (88.9%) completely agreed with the statement that they were satisfied with the mobile app.

### Usability

Among participants who provided survey data, most completely agreed 13/18 (72.2%) that the app was easy to learn. Most completely agreed 12/18 (66.7%) that navigating the app was clear and understandable. About half completely agreed 10/18 (55.6%) that logging food through the app was easy. A few participants noted challenges with using the app, such as difficulty finding specific food items or not having enough food choices in the app.

### Informal caregiver involvement

Among participants who provided survey data, a little more than a quarter 5/18 (27.7%) reported that their informal caregiver assisted them with logging their food intake.

### Percent of caloric goal obtained

**Fig. 1:**
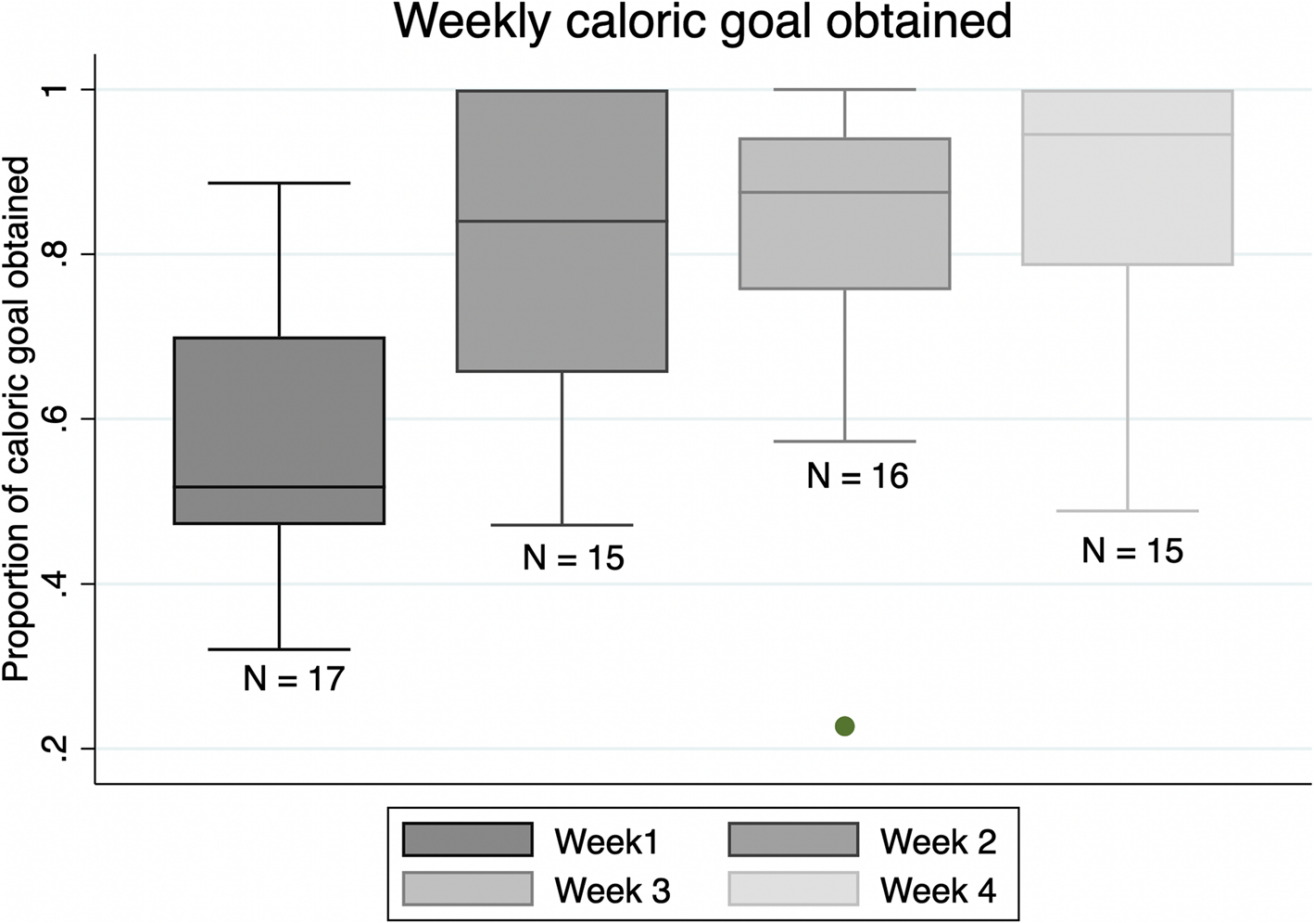
Weekly caloric goal attainment among study participants

Among participants with complete data (*n* = 15) (24/30 days of data), the percent caloric goal obtained was 82.4% (IQR: 21.7) over the 30-day period. During week 1, among participants with complete data (*n* = 17) (5/7 days of data), the percent caloric goal obtained was 51.8% (IQR: 26.5) (Fig. 1). During week 2, among participants with complete data (*n* = 15), the percent caloric goal obtained was 84.0% (IQR: 34.4). During week 3, among participants with complete data (*n* = 16), the percent caloric goal obtained was 87.5% (IQR: 18.6). During week 4, among participants with complete data (*n* = 15), the percent caloric goal obtained was 94.5% (IQR: 24.0).

## Discussion

Overall, the goal of this study was to assess the feasibility, acceptability, usability, and preliminary efficacy of a remote nutrition counseling intervention to enhance nutrition recovery after HPB surgery. Our study found that the intervention met a priori feasibility and acceptability benchmarks. Most participants were retained over the study period, were highly engaged with the mobile app and the dietitian visits, and most reported high satisfaction with the intervention. Most participants found the app easy-to-use; however, a few participants reported challenges with locating food items within the app. In terms of preliminary efficacy, participants struggled meeting their daily caloric goals during the first week after surgery; however, most participants began achieving their caloric goals by week 2 and were able to maintain their caloric goals through weeks 3 and 4.

Prior studies suggest that the transition from hospital to home after pancreatectomy and hepatectomy is challenging for many patients with cancer [33, 34, 42]. Patients are expected to make complex dietary changes (e.g., tracking calorie intake) and modify behavior (e.g., small, frequent meals). Few patients receive support, however, for managing these changes. The current study adds to the literature by suggesting that nutrition counseling coupled with digital tools, such as a mobile app to track food intake, is feasible and acceptable among this patient population. For example, most patients (79%) logged food intake for more than 80% of study days and nearly all participants (95%) attended all three dietitian visits during the study period. These findings suggest that there is patient demand for enhanced nutrition monitoring after HPB surgery. However, there are no published accounts of mobile app monitoring of post-operative caloric intake. This study is the first to our knowledge to use mobile apps for this purpose.

One of the key challenges of delivering a digital intervention is developing a tool that is easy to learn and use across a wide array of patients. Our study found that most but not all patients agreed the app was easy to learn, navigate, and use for logging food intake. Several challenges were also identified, such as difficulty finding specific food items or not having enough food choices within the app. Our dietitian noted that several participants reported challenges when the exact food item (e.g., specific brand of yogurt) was unavailable in the app. The dietitian clarified to patients that an approximation (e.g., selecting a brand of yogurt that is available) is sufficient. In addition to optimizing patient instructions, future studies may need to assess digital literacy (e.g., eHealth literacy scale) [43] to determine whether usability varies based on pre-existing digital literacy. Our study found that about a quarter of patients required informal caregiver assistance to help enter food intake data in the mobile app. Future studies might add an informal caregiver training component to help informal caregivers also learn how to use the app.

Our study found that caloric intake trended upwards over the 30-day period. Most participants struggled with food intake in the first week post-discharge but quickly recovered during weeks 2–4. Our intervention started dietitian visits 2 weeks post-surgery. Future studies might consider starting dietitian visits 1 week after surgery, when patients have the most difficulty meeting their caloric intake goals. The increase in caloric intake over time may also be explained by the type of surgeries examined. For example, at the time of first surgical follow-up, most patients undergoing post-Whipple/total pancreatectomy advance from a low-fat diet to a regular diet.

Remote monitoring of caloric intake has the potential to be used as a clinical indicator for HPB surgery patients, which can be intervened upon via phone consultation, secure messaging or expedited clinic follow up [44–47]. Highlighting this potential, 2 of 4 patients in our study with less than 80% app compliance presented back to the hospital with significant complications including post-operative pancreatic hemorrhage and sepsis, respectively. Implementation of monitoring and counseling has the potential to extend the reach of dietitians amongst a population who many believe should receive universal nutritional screening [48, 49]. While more research is necessary, nutrition monitoring mobile apps have potential for both the pre-habilitation and post-operative setting for cancer patients and other patients receiving complex surgeries. Nutritionally replete patients benefit from fewer perioperative complications, shorter hospitalizations, higher completion of multimodality therapy, and improved survival [50, 51].

## Limitations

This study has several limitations. First, the study was conducted at an National Cancer Institute Designated Comprehensive Cancer Center, which may have resources that are not available in all oncology settings (e.g., onsite registered dietitian). Second, this study was designed to assess feasibility, acceptability, and app usability and was not powered to evaluate efficacy. A larger trial is needed to determine the efficacy of the intervention. Findings from this study suggest that the intervention is feasible and could be tested in a larger trial. Third, the study was limited to participants who speak English. The usability of the app may differ for languages other than English. Future studies should test the usability of the mobile app in Spanish speakers and other languages (e.g., Arabic) available through the MyPlate app [52].

## Conclusion

Malnutrition is a common and devastating consequence of cancer and surgical treatment, such as HPB surgery. Despite its severity, there are limited interventions addressing malnutrition after surgery. Patient dietary self-monitoring through a mobile app, combined with dietitian counseling, in the post-operative period after HPB surgery appears to be feasible and acceptable. Most patients were able to achieve their caloric goals within 2–4 weeks of surgery, suggesting this intervention could improve patient outcomes. Further studies are needed to test this approach in a larger efficacy trial.

## Data Availability

The authors will make de-identified data available upon request with the establishment of a data sharing agreement. Requests can be sent to kea.turner@moffitt.org.

## Abbreviations

HPB: Hepatopancreaticobiliary
IQR: Interquartile range
